# Telocytes in the human ascending aorta: Characterization and exosome‐related KLF‐4/VEGF‐A expression

**DOI:** 10.1111/jcmm.16919

**Published:** 2021-09-25

**Authors:** Thomas Aschacher, Katy Schmidt, Olivia Aschacher, Eva Eichmair, Ulrike Baranyi, Bernhard Winkler, Martin Grabenwoeger, Andreas Spittler, Florian Enzmann, Barbara Messner, Julia Riebandt, Guenther Laufer, Michael Bergmann, Marek Ehrlich

**Affiliations:** ^1^ Department of Cardio‐Vascular Surgery Clinic Floridsdorf and Karl Landsteiner Institute for Cardio‐Vascular Research Vienna Austria; ^2^ Centre for Anatomy and Cell Biology Medical University of Vienna Vienna Austria; ^3^ Department of Plastic, Reconstructive and Aesthetic Surgery Medical University of Vienna Vienna Austria; ^4^ Cardiac Surgery Research Laboratory Department of Cardiac Surgery Medical University of Vienna Vienna Austria; ^5^ Department of General Surgery Medical University of Vienna Vienna Austria; ^6^ Department of Vascular Surgery Medical University of Innsbruck Innsbruck Austria

**Keywords:** adventitia, CD34, exosome, human thoracic aorta, KLF‐4, PDGF‐A, telocytes, transmission electron microscopy, VEGF‐A

## Abstract

Telocytes (TCs), a novel interstitial cell entity promoting tissue regeneration, have been described in various tissues. Their role in inter‐cellular signalling and tissue remodelling has been reported in almost all human tissues. This study hypothesizes that TC also contributes to tissue remodelling and regeneration of the human thoracic aorta (HTA). The understanding of tissue homeostasis and regenerative potential of the HTA is of high clinical interest as it plays a crucial role in pathogenesis from aortic dilatation to lethal dissection. Therefore, we obtained twenty‐five aortic specimens of heart donors during transplantation. The presence of TCs was detected in different layers of aortic tissue and characterized by immunofluorescence and transmission electron microscopy. Further, we cultivated and isolated TCs in highly differentiated form identified by positive staining for CD34 and c‐kit. Aortic‐derived TC was characterized by the expression of PDGFR‐α, PDGFR‐β, CD29/integrin β‐1 and αSMA and the stem cell markers Nanog and KLF‐4. Moreover, TC exosomes were isolated and characterized for soluble angiogenic factors by Western blot. CD34^+^/c‐kit^+^ TCs shed exosomes containing the soluble factors VEGF‐A, KLF‐4 and PDGF‐A. In summary, TC occurs in the aortic wall. Correspondingly, exosomes, derived from aortic TCs, contain vasculogenesis‐relevant proteins. Understanding the regulation of TC‐mediated aortic remodelling may be a crucial step towards designing strategies to promote aortic repair and prevent adverse remodelling.

## 1 INTRODUCTION

Telocytes (TCs) are a recently defined interstitial cell type, morphologically characterized by a small oval cell body and a variable number of thin, long branching processes called telopodes (Tps).^1^, ^2^ These Tps comprise alternating regions of podoms (including mitochondria, endoplasmic reticulum and caveolae) and thin podomeres.^3^ TCs have been found in a wide range of tissues^2^, ^3^, ^4^, ^5^, ^6^, ^7^, ^8^ including the heart^9^, ^10^, ^11^ and the vascular system.^12^, ^13^, ^14^, ^15^ TCs were detected in close relation to blood vessels, nerve endings and smooth muscle cells (SMCs)^6^, ^12^, ^16^ and participate in inter‐cellular signalling, tissue remodelling, renewal and regeneration.^2^, ^9^, ^17^

With respect to the peripheral vascular system, TCs were only described in arterioles, venoules and capillaries.^15^, ^18^ In medium‐sized arteries, the number of TCs was increased in the adventitial layer after vascular injury in rats.^12^ Moreover, TCs appeared to be adherent in the endothelial layer of pig coronary arteries.^14^ Although Li H. et al. excluded the possibility of TCs in bigger vessels due to their reduced tolerance to turbulent blood flow,^14^ they were located in the adventitial layer of the aortic arch of mice.^13^

To our knowledge, TCs have not been described in the human aorta in literature up to now. A potential contribution of TC in aortic tissue homeostasis could be of great interest as regeneration of the aorta would be of high clinical relevance. Its misbalance is considered to lead to aortic aneurysms, a potentially fatal disease when resulting in dissection or rupture.

The human ascending thoracic aorta (HTA) differs embryologically and genetically from the aortic arch and plays a crucial role in the development of aortic dilatation—from aneurysm formation to lethal dissection.^19^, ^20^, ^21^ Therefore, the presence of TCs in the HTA, with the ability for tissue remodelling and regeneration, should be of specific interest. The results could give vital information for the development of HTA pathologies.

TC is known to have tissue‐specific makers. In this line, only the group of Yanyan Li identified specific molecular markers, for vascular TC, which were described in middle‐sized arteries.^12^ They found the mesenchymal cell marker vimentin and the hematopoietic cell marker CD34 in TC. Little more analyses were done to identify the immunophenotype of aortic TCs.

The aim of this study was to gather first insights into the existence, characteristics and distribution of TCs in the different layers of HTA by transmission electron microscopy and light microscopy. The study was also designed to investigate the specific cell/stem cell markers of aortic TCs. We also establish the isolation of TCs and characterized TC‐derived microparticles.

## 2 MATERIALS AND METHODS

### 2.1 Human specimen

Twenty‐five human non‐pathological aortic tissue samples were obtained during heart transplantation from donors’ hearts. Samples were only taken if the aorta had to be shortened to fit during implantation. After receiving the specimens, aortic tissue was washed three times with sterile PBS (PAA Laboratories, Inc., Austria) to remove blood residues. Tissue was cut into pieces and processed as following: one piece was snap‐frozen and stored in liquid nitrogen, one piece was fixed in 4.5% formalin, and another one in 2.5% glutaraldehyde and the remaining part was subjected to cell isolation. This study was approved by the ethical committee of the Medical University of Vienna (EK 1280/2015). Written informed consent was obtained from all patients prior to inclusion in the study. The investigation conformed to the principles that are outlined in the Declaration of Helsinki regarding the use of human tissue.

### 2.2 Isolation and fluorescence‐activated cell sorting (FACS) of aortic telocytes

Aortic TCs were isolated from healthy human ascending aortic tissue obtained from healthy heart donors during heart transplantation as previously described.^11^ Samples were collected in Ringer's lactate solution (B. Braun, Austria) during heart transplantation and processed within 60 min after surgery. Human aortic samples were collected into sterile tubes containing Dulbecco's modified Eagle's medium (DMEM), supplemented with 2% foetal bovine serum (FBS), 1.5 mM HEPES as well as 200 IU/ml penicillin and 200 UI/ml streptomycin (medium and all supplements were obtained from Gibco/Life Technologies Ltd., Pailey, UK), placed on ice and transported to the cell culture laboratory. The aortic samples were dissected and minced into small pieces of about 1 mm^3^, subsequently washed and incubated for 3 hours, at 37℃, with collagenase type IV (Gibco), elastase (porcine pancreas, Calbiochem/Merck, Germany) in DMEM supplemented with 10% FBS, 1,5 mM HEPES, 100 IU/ml penicillin and 100 IU/ml streptomycin. The dispersed cells were separated from non‐digested tissue by filtration through a cell strainer (100 µm), collected by centrifugation of the filtrate at 250 g for 10 min, at RT (22℃) and resuspended in culture medium. Cells were seeded into 25‐cm^2^ plastic culture flasks (BD Falcon, San Jose, CA, USA). The supernatant was collected after 60 and 90 minutes and re‐plated in 24‐well chambers with culture medium containing high‐glucose DMEM (HG‐DMEM), 10% FBS and 100 IU/ml penicillin (Sigma‐Aldrich Inc., MO, USA) and 100 IU/ml streptomycin (Sigma‐Aldrich). Cultivation of cells was performed in gelatine‐coated polystyrene culture flasks, and medium was changed every second day. TCs attached after plating for 45–60 min. After cultivation for 48h, TC proliferation reached the logarithmic growth phase. Following 72 h of primary culture, single adherent cells displayed typical TC morphology. Cells were maintained at 37℃ in humidified atmosphere (5% CO2 in air) until becoming semi‐confluent (usually 4 days after plating) when the cells were detached using 0.25% trypsin (Sigma‐Aldrich) and 2 nM EDTA (Sigma‐Aldrich) and re‐plated at the same density of 5 x 4 cells/cm^2^. The morphology of TCs was observed, and cells were imaged with a phase‐contrast microscope (Olympus CKX41 with Olympus SC‐20 camera, Olympus Life Science, Vienna, Austria).

For CD34/c‐kit‐specific cell sorting of isolated aortic TCs, cultured TCs were collected in FACS buffer (PBS including 0.1% FBS), and 25mM HEPES was added to the FACS buffer to prevent it from becoming basic and maintain the pH between 7.0 and 8.0, and 1mM‐5mM EDTA to the buffer to prevent formation of aggregates. Cells were stained with 1x of the antibody concentration used for immunocytochemistry, followed by appropriate secondary antibody (see Table 1). Cells were resuspended at a concentration of 2‐3x10^7^ cell/ml. Immediately before sorting, cells were filtered through a 70‐µm mesh filter to prevent clogging and collected in HG‐DMEM supplemented with 30% FBS afterwards. Collected cells were divided, one part was analysed directly by Western blot and the second part was cultivated in standard culture medium. Cell sorting was performed with the BD FACSAria™III Fusion (Software: BD FACSDiva Version 8.0.2).

**TABLE 1:** List of antibodies and working dilutions used in this study

Antibody name	Source	Cat.no	Working Dilution WB	Working Dilution ICC	Working Dilution IF
Primary antibodies					
c‐kit (CD117)	Santa Cruz	sc365504	1:450	1:100	1:100
c‐kit (CD117)	Abcam	ab32363	1:200	1:200	1:200
c‐kit (CD117)	BioTechne	AF1356	‐	1:75	‐
PDGFR‐α	Santa Cruz	sc398206	1:400	1:300	1:100
PDGFR‐β	Abcam	ab69506	1:600	1:50	1:300
KLF−4	Abcam	ab75486	1:50	1:100	1:100
VEGF‐A	Abcam	ab1316	1:100	1:500	1:100
CD90	Dianova	15630/01	1:500	1:50	1:200
CD34 (B6)	Santa Cruz	sc74499	1:400	1:500	1:100
αSMA	Abcam	ab5694	1:500	1:200	1:500
Vimentin	Santa Cruz	sc5565	1:400	1:1000	1:200
CD133 (EPR20980‐104)	Abcam	ab216323	1:800	1:100	1:1000
Nanog	Novus	NB110‐40660	‐	1:200	‐
CD29/integrin β−1	BDPharmigen^TM^	556048	‐	1:50	‐
SM‐calponin	Santa Cruz	sc58707	‐	1:100	‐
CD31	Dako	M0823	‐	‐	1:40
Secondary antibodies					
AF488GAR	Molecular probes	A11034	‐	1:1000	1:500
AF546GAM	Molecular probes	A11030	‐	1:1000	1:1000
AF488GAM	Molecular probes	A11029	‐	1:1000	1:1000
AF546GAR	Molecular probes	A11035	‐	1:1000	1:1000
AF488DAG	Molecular probes	A31576	‐	1:500	1:500
Isotype‐control					
Purified mouse IgG1	BDPharmigen	550878	‐	4 µg/ml	4 µg/ml
Purified rabbit IgG	Abcam	Ab27478	‐	1.89 µg/ml	1.89 µg/ml

Note

Abcam, Cambridge, UK; BD Pharmigen^TM^ (BD Biosciences), San Jose, CA; Santa Cruz Biotechnologies, Inc., TX, USA; Invitrogen Molecular Probes, Thermo Fisher Scientific, Massachusetts, USA; Novus Biologicals, LLC, CO, USA; Dianova GmbH, Hamburg, Germany.

### 2.3 Isolation and cell sorting by Dynabeads of fibroblasts and vSMCs

Cell isolation and sorting of CD90^+^ fibroblasts and α‐SMA^+^vSMC were done as described above. Except, after filtration the cells were seeded and cultured in in DMEM supplemented with 10% FBS, 1,5 mM HEPES, 100 IU/ml penicillin and 100 IU/ml streptomycin. After reaching cell confluence, cells were harvested and proceeded with FACS method as described above. Purity estimation was done by Western blot.

### 2.4 Immunocytochemical staining and microscopy

Cells were grown on 8‐chamber slides (Nunc^®^ Lab‐Tek^®^ Chamber Slide™, Sigma‐Aldrich) for 2 days, washed with PBS (Thermo Fisher Scientific, Massachusetts, USA) and fixed in 4% paraformaldehyde for 10 min, followed by permeabilization in 0.1% saponin and blocked with PBS containing 1% bovine serum albumin (BSA), 10% goat serum and 0.3 M glycine for 1h at 37℃. Samples were incubated with 2–5 µg of primary antibody O/N with the listed working dilutions (Table 1), followed by incubation with an appropriate secondary antibody and mounted in VECTASHIELD mounting medium including 0.5 µg/ml DAPI (VECTASHIELD; VectorLabs, Burlingame, CA). Negative controls were obtained following the same protocol, but omitting the primary antibodies, and the usage of purified anti‐mouse and anti‐rabbit IgG (Abcam, Cambridge, UK). For confocal microscopy, we used a LSM700 Meta microscopy laser system, the appropriate filters and a ZEN 2010 microscopy system (Zeiss, Inc. Jena, Germany).

### 2.5 Immunohistochemical and immunofluorescence staining

Twenty‐five aortic tissue samples were fixed in 4% PBS‐buffered formaldehyde at 4℃ for a minimum of 24 hours. The samples were embedded in paraffin. Sections were deparaffinized with xylene and rehydrated in a descending series of ethanol (96%, 80%, 70% and 50%). Following heat‐induced antigen retrieval with citrate‐buffer (pH 6), the sections were blocked with 10% goat serum, 1% BSA, 0.3 M glycine and 0.1% Tween‐20 in PBS^−/−^ at RT for 60 min. The antibody incubations corresponded to ICC staining protocol. The density of TCs was calculated as the mean of total number of TCs/total number of DAPI stained nuclei of 23 human specimens. For haematoxylin and eosin (H&E) staining, sections of aortic tissue were pre‐treated as for immunohistochemistry. Haematoxylin solution was added to the sections for 8 min at RT, followed by 1% acid alcohol for 30 sec, before counterstaining with eosin‐phloxine solution for 1 min at RT. Sections were then dehydrated and cleared with xylene and mounted. H&E stain was applied to analyse primary localization of TCs and general tissue morphology.

### 2.6 Transmission electron microscopy

Samples of the aortic wall of approx. 2 cm^2^ were fixed immediately after surgery in 2.5% glutaraldehyde. After 6 hrs, samples were cut into smaller pieces of 1 mm^3^ and washed three times in 0.1 M cacodylate buffer. The secondary fixation was carried out either for 2 h in 2% osmium tetroxide / 0.1 M cacodylate buffer or for 2 h in 1% reduced osmium tetroxide, both at room temperature. Dehydration and embedding in Epon resin followed standard procedures. Ultrathin sections (70 nm) were cut with a Reichert UltraS microtome and contrasted with uranyl acetate and lead citrate. Images were acquired with a FEI Tecnai20 electron microscope equipped with an 4K Eagle CCD camera and processed using the Adobe Photoshop software package.

### 2.7 Microvesicle and exosome isolation

Microvesicle and exosome isolation was performed as previously described with minor modifications.^22^ Briefly, cells were grown in FCS‐free culture medium for 24 h. The cell suspension was centrifuged at 480g at 4℃ for 5 min to remove any intact cells, followed by a 3200 *g* spin at 4℃ for 20 min to remove dead cells. To isolate shed microvesicles (MVs), the supernatant was centrifuged at 10,800 g at 4℃ for 20 min in an Optima L80 ultracentrifuge with a SW41Ti rotor (Beckman Coulter, Mississauga, Canada). The pellet, containing sMV, was washed once with PBS^−/−^ and ultracentrifuged at 10,800 *g* for 30 min. The pellet was dissolved in fresh medium for immediate use or stored at −80℃ for Western blot analysis. The remaining culture medium was transferred to ultracentrifuge tubes and sedimented at 110,000 *g* at 4℃ for at least 75 min. The supernatant constituting exosome‐free medium was removed, and the pellets containing exosomes plus proteins from media were resuspended in PBS. The suspension was centrifuged at 100,000 *g* at 4℃ for at least 60 min to collect final exosome pellets. The quality of exosomes was confirmed by qNano analysis (Izon instrument, UK). Protein content of the exosome pellet was quantified using the Bradford protein assay kit (Bio‐Rad, Hercules, CA).

### 2.8 RNA isolation and real‐time PCR (RT‐qPCR)

RNA isolation and RT‐qPCR were performed in isolated and CD34^+^/c‐kit^+^ sorted aortic TCs. RNA was isolated using TRIzol (PeqGOLD TriFast, Peqlab, VWR, Vienna, Austria) followed by purification with the E.Z.N.A. Microelute Total RNA Kit (Omega Bio‐Tek, VWR, Vienna, Austria), including the optional DNA digestion step (RNase‐free DNase I Set, Omega Bio‐Tek, VWR, Vienna, Austria) according to manufactures’ instructions. RNA was reverse transcribed using the QuantiTect Reverse Transcription Kit (Qiagen, Hilden, Germany). For RT‐qPCR, cDNA and gene‐specific primers were used with the Maxima® SYBR Green/ROX RT‐qPCR MM (2X) (Fermentas, Fisher Scientific, Austria) or the GoTaq RT‐qPCR Master Mix (Promega, Mannheim, Germany). Samples were normalized to the geometric mean of two reference genes (GAPDH, RPLP0). Primer sequences are listed in Table 2.

**TABLE 2:** List of used RT‐qPCR primers

Target	Forward‐primer	Reverse‐primer
PDGFR‐α	GACTTTCGCCAAAGTGGAGGAG	AGCCACCGTGAGTTCAGAACGC
PDGFR‐β	AGGACAAACCGTACCTTGGGTGACT	CAGTTCTGACACGTACCGGGTCTC
CD34	CACCCTGTGTCTCAACATGG	GGGAGATGTTGCAAGGCTAG
CD90	TTTAGTTATAGTTTTGGGAAAGGATAT	CCACCTCCTCCCTCTATT
αSMA	AACTGGTATTGTGCTGGACTCTGG	CACGGACGATCTCACGCTCAG
CD31	GCTCTCTTGATCATTGCG	GAGGACACTTGAACTTCC
c‐kit (KIT)	TCATCGAGTGTGATGGGAAA	GGTGACTTGTTTCAGGCAACA
SM‐calponin	CTGGCTGCAGCTTATTGATG	CTGAGAGAGTGGATCGAGGG
CD29/integrin β−1	AGAAGCTCAAGCCAGAGG	GCATCTGTGGAAAACACC
RPLPO (housekeeping gene)	AGCCCAGAACACTGGTCTC	ACTCAGGATTTCAATGGTGCC
GAPDH (housekeeping gene)	TGCACCACCAACTGCTTAGC	GGCATGGACTGTGGTCATGAG

### 2.9 Western blot analysis

Whole cell lysate was prepared by scraping cells in 250 μl of ice‐cold‐modified RIPA buffer [50 mM Tris‐Cl (pH 7.4), 150 mM NaCl, 1 mM EDTA, 1% NP‐40, 0.25% Na‐deoxycholate, 1mM PMSF, 10 μg/ml aprotinin, 10 μg/ml leupeptin, 1 mM Na_3_VO_4_ and 1 mM NaF].^23^ The lysate was rotated 360° at 4℃ for 1h followed by centrifugation at 12,000 *g* at 4℃ for 10 min to clear the cellular debris. Total protein was quantified using the Bradford Protein Assay Kit (Bio‐Rad). Equal amounts of protein were resolved on an SDS‐polyacrylamide gel and transferred to a nitrocellulose membrane. The previously described antibodies were used for Western blot analysis. Immunodetection was performed by blocking the membranes for 1h in TBS buffer [20mM Tris‐Cl (pH 7.5), 137mM NaCl, 0.05% Tween‐20] containing 5% powdered non‐fat milk followed by addition of the primary antibody (as indicated) in TBS buffer for 2 h at RT. Specifically bound primary antibodies were detected with peroxidase‐coupled secondary antibodies and developed by enhanced chemiluminescence (ECL system, Amersham Pharmacia Biotech Inc., Arlington Heights, IL) according to manufactures’ instructions. All experiments were performed at least three times using independent biological replicates.

#### 2.9.1 Morphometric and statistical analysis

Morphometric studies were performed in a double‐blinded manner. A minimum of 10 randomly selected sections of aortic samples were analysed by two independent investigators. Data are expressed as mean ±SD and were analysed by using Student's *t* test for independent samples. *p* < 0.05 was considered statistically significant. Statistical analyses were performed with GraphPad Prism version 6 (GraphPad Software, CA, USA) and SPSS 25.0 software (2020, SPSS Inc, Chicago, USA).

## 3 RESULTS

### 3.1 Histological analysis of aortic tissue

**FIGURE 1:**
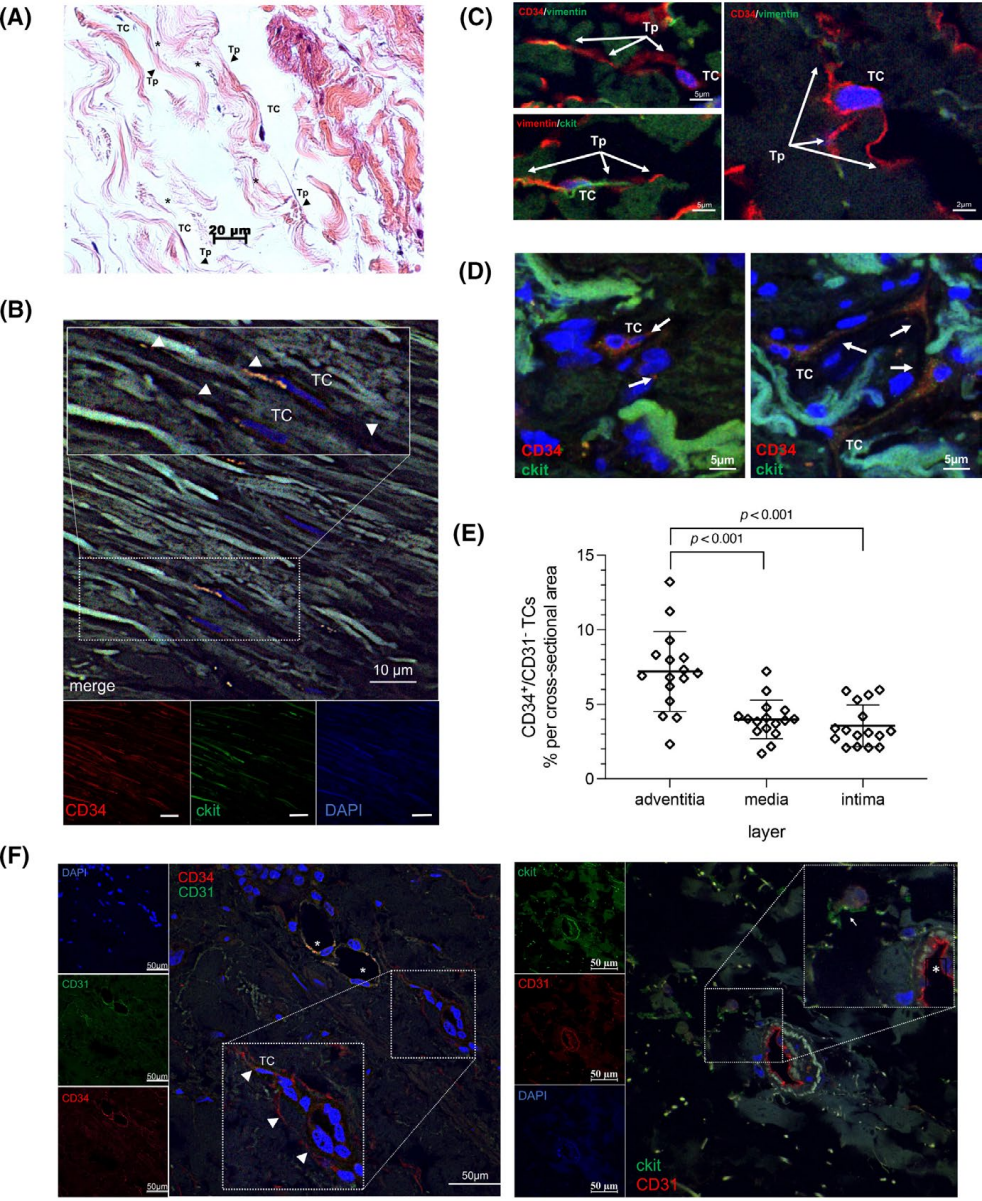
Telocytes (TC) were identified in human thoracic aorta. (A) Tunica adventitia. H&E. Telocytes with an oval‐stretched body and long, thin processes (narrow) oriented parallel to collagenous fibres (asterisk). (B) Tunica media. CD34 (red), c‐kit (green) and cell nuclei (DAPI, blue) labelling. CD34^+^/c‐kit^+^ TCs were detected embedded inbetween collagen fibres with highly by double positively stained of TC processes (narrows). The magnification shows two double‐positive TCs (arrowheads point to Tps). (**C)** TCs were stained as indicated top left in each image with combinations of c‐kit (green), vimentin (green) and CD34 (red). Shapes of TCs: i) piriform—top left; ii) spindle—bottom left; iii) triangle—right. Classification depending on number and orientation of Tps. (D) Presumed communicative network of CD34^+^/c‐kit^+^ TCs with other cells via Tps (arrows) in the tunica adventitia (left and right images). CD34 (red), c‐kit (green) and cell nuclei (DAPI, blue). TC, telocyte body; Tp, telopodes. (E) Statistical analysis of CD34^+^/CD31^−^ TC cell counts in the individual layers of aortic tissue. (F) CD31^+^ and CD34^+^ nucleated cells are present in the intima of vasa vasorum (asterisk, left panel). CD34^+^/CD31^−^ cells (left panel) and CD31^−^/c‐kit^+^ cells (right panel) with TC‐specific features (arrowheads point to telopodes) were found adjacent region of adventitia. CD31 (red), CD34 (green, left panel) or c‐kit (green, right panel) and cell nuclei (DAPI, blue). TC, telocyte body

The presence and distribution of TCs was assessed in HTA tissue. For the initial histological analysis of potential presence of TCs in HTA, the surgical specimens were stained by toluidine blue‐ and H&E as described previously.^3^ Morphological analysis of HTA showed cells with potential ‘TC‐typical’ cell features mainly localized in the adventitial layer (Figure 1A).

To provide evidence of TCs in human aortic samples, we performed immunofluorescence staining. We identified CD34^+^/c‐kit^+^ double‐positive cells, as well as CD34^+^/vimentin^+^ double‐positive cells, and vimentin^+^/c‐kit^+^ double‐positive cells in HTA (Figure 1B–1F). As observed in other organs,^1^, ^4^, ^24^, ^25^, ^26^, ^27^ TCs showed the typical cell body shape, oval or triangular, containing a heterochromatic nucleus and presenting thin long protrusions (podomeres) with dilated segments (podoms). A further analysis of tissue samples showed spindle‐shaped, polygonal and stellate cells with long thin processes in the transition of the adventitial to the medial aortic layer (Figure 1B and Table 3), independent of the donor's age and baseline characteristics.

**TABLE 3:** Differences in CD34^+^/c‐kit^+^ TC types depending on location found in human aortic tissue

Parameter	Location of TCs		
	T. intima	T. media	T. adventitia
Cell shape	Oval	Spindle‐shape	Oval‐trigeminal
Cell diameter, µm	3.64 ± 1.1µm	2.3 ± 0.7µm	4.32 ± 1.2µm
Nucleus shape	oval	irregular	Oval with nucleolus
Nucleus diameter, µm	1.12 ± 0.5µm	1.67 ± 0.3µm	2.34 ± 0.8µm
Number of processes	2	2–3	2–4
c‐kit expression	+	+	++

Note

+, weakly positive; ++, highly positive.

We classified the three layers of aortic tissue, namely the tunica intima, tunica media and tunica adventitia, to pinpoint the differences in TC presence and features depending on their location in HTA (Figure 1C and Table 3). Most recently, Billaud et al. described a CD34^+^ progenitor cell population associated with the vasa vasorum in the human adult aorta,^28^ whereby a subset of these cells co‐expressed the endothelial cell marker CD31 and where described for the endothelium of vasa vasorum, which should be distinct from CD34^+^/CD31^−^ TCs. Therefore, we performed a CD34/CD31 double staining, as well as a CD31/c‐kit double staining, and demonstrate a TC specificity by the lack of endothelial marker CD31 expression (Figure 1E and 1F). However, the adventitia showed the highest density of CD34^+^/CD31^−^ TC, compared to the medial or intimal layer (Figure 1E).

### 3.2 Ultrastructural characteristics of aortic TCs

**FIGURE 2:**
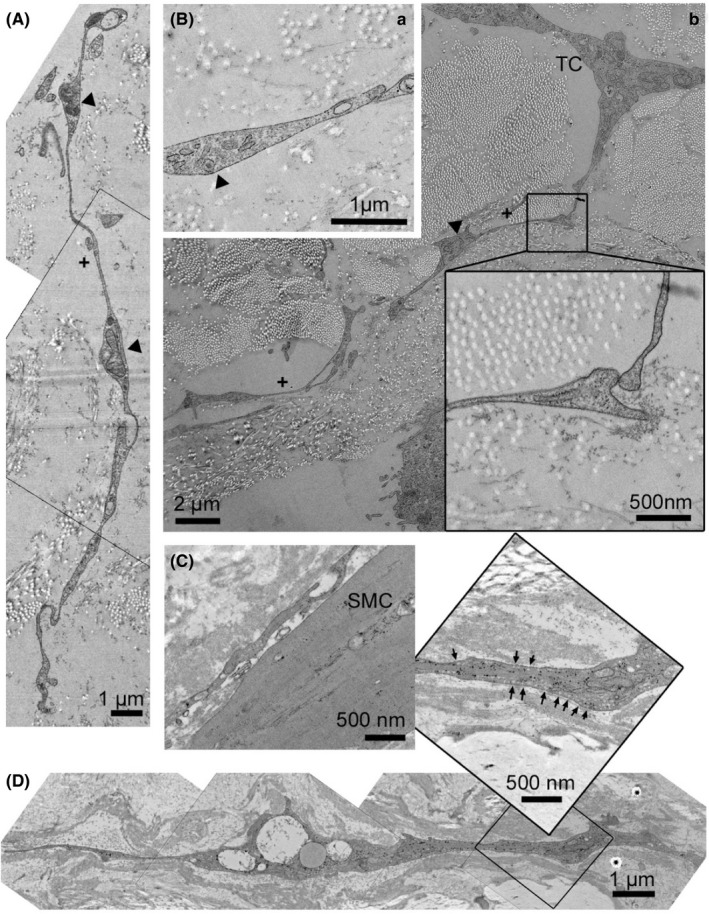
Transmission electron microscopy of aortic telocytes (TC). (A–B) TCs embedded in loosened tunica adventitia between collagen fibres and cell‐free extracellular matrix. (A) Telopodes consist of alternating podomeres (arrowheads) with less than 0.2 µm thin segments (plus). (B) Typical characteristics of TCs in this population are extremely long cell processes (b) and the formation of cell‐cell contacts between them (a and inset). (C–D) TCs in close connection to aortic smooth muscle cells (SMC) and elastic fibres. (C) A direct contact between a telopode and an SMC is shown. (D) One characteristic of the second population is a high number of internal vesicles (arrows) as seen in the magnified detail of the image above

Telocytes were studied by transmission electronic microscopy (TEM) in healthy aortic tissue. TEM analysis represents the gold standard of TC identification and characterization.^1^, ^18^, ^25^ In ascending thoracic aortic tissue, TCs with long Tps were clearly identifiable (Figure 2A and 2B). Tps were detected based on their discontinuous segments with alternating podoms and podomeres (Figure 2A). We focussed on the adventitial layer since it presented the highest number of TCs with the most typical structural features.^2^ The observed TC features include the following: (i) elongated spindle‐shaped cells (Figure 2Bb), (ii) two to four ultrathin processes from fifty to several hundreds of microns of length (Figure 2B and 2D) and (iii) processes containing thin fibril‐like segments and vacuoles (vesicles; multivesicles or exosomes) (Figures 2C, 2D, and 5B). TEM revealed two TC populations. One population presented with spindle‐shaped cells and was found to be embedded in fibrillin and collagen. The Tps were extremely long with a rare count of vacuoles or multivesicular bodies (MVs) (Figure 2B). TCs in this population were frequently found to form a TC network (Figure 2Ba and inset). In contrast, the second population of TCs showed a very close relation to SMCs (Figure 2C). These cells appeared to have more and shorter but wider processes than the spindle‐shaped cells. This population showed a high number of vesicles located in and around Tps (Figures 2D and 5B).

### 3.3 Isolation and identification of aortic TCs

**FIGURE 3:**
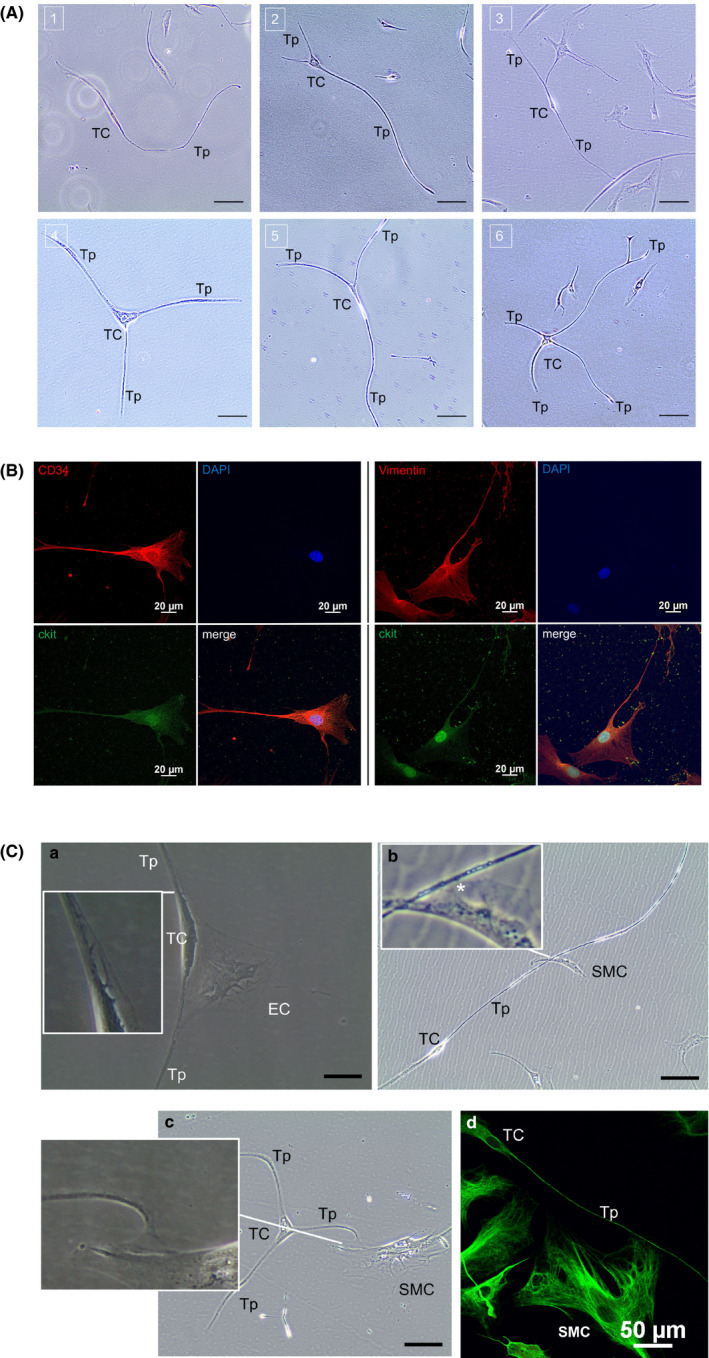
Characterization of isolated aortic TCs. (A) Different shapes of aortic TCs shown by light microscopy eight days after isolation. Piriform spindle‐shaped TCs (1, 3); triangular‐shaped TCs (2, 4, 5); stellate‐shaped TC (6). Scale bar: 20 µm. (B) CD34/c‐kit (left) and vimentin/c‐kit (right) immunofluorescence staining. TCs were identified as double‐positive cells with CD34 and vimentin or c‐kit, respectively. Nuclei were counterstained with DAPI (blue). Scale bar: 20 µm. (C) Indication of cell‐cell communication. Direct cell‐cell contacts between TCs/Tps and co‐cultivated ECs (1, 3 and 4) or SMCs (2). The areas of cell connections are shown in enlarged sections for 1–3 and further pointed out in 2 (asterisk). An immunofluorescence stain against vimentin (green) is presented in (4). Scale bar: 1, 20 µm; 2–4, 50 µm. EC, endothelial cell; SMC, smooth muscle cell; TC, telocyte body; Tp, telopode

Primary TC cultures exhibit a characteristic morphology and can easily be distinguished from fibroblasts, SMC and epithelial cells by phase‐contrast microscopy^11^, ^29^ (Figure 2C). TCs were isolated from healthy HTA tissue as described previously^11^ and showed long and thin podomeres as well as dilated podoms extending from a variable sized and shaped cell body (Figure 3A). In the first three days of culture, TC cell bodies were primarily piriform‐shaped depending on the number of Tps. After 5–7 days, we observed an increasing number of spindle‐ and triangular‐shaped TCs. To test for previously described TC markers,^2^, ^15^, ^27^, ^29^ primary aortic cells were stained by immunofluorescence (Figure 3B). Further, we observed that TCs started to form a connective ‘network’ between TCs and vSMC, or other TCs (Figure 3C). The length of the plasma membrane stretches involved in cell‐cell connections varies from dozens to hundreds of micrometres. TCs were exclusively interacting via their Tps with other cells and showed an increase in small vacuoles in the cytoplasm of the connecting region.

### 3.4 Identification of specific aortic TC markers by immunofluorescence

**FIGURE 4:**
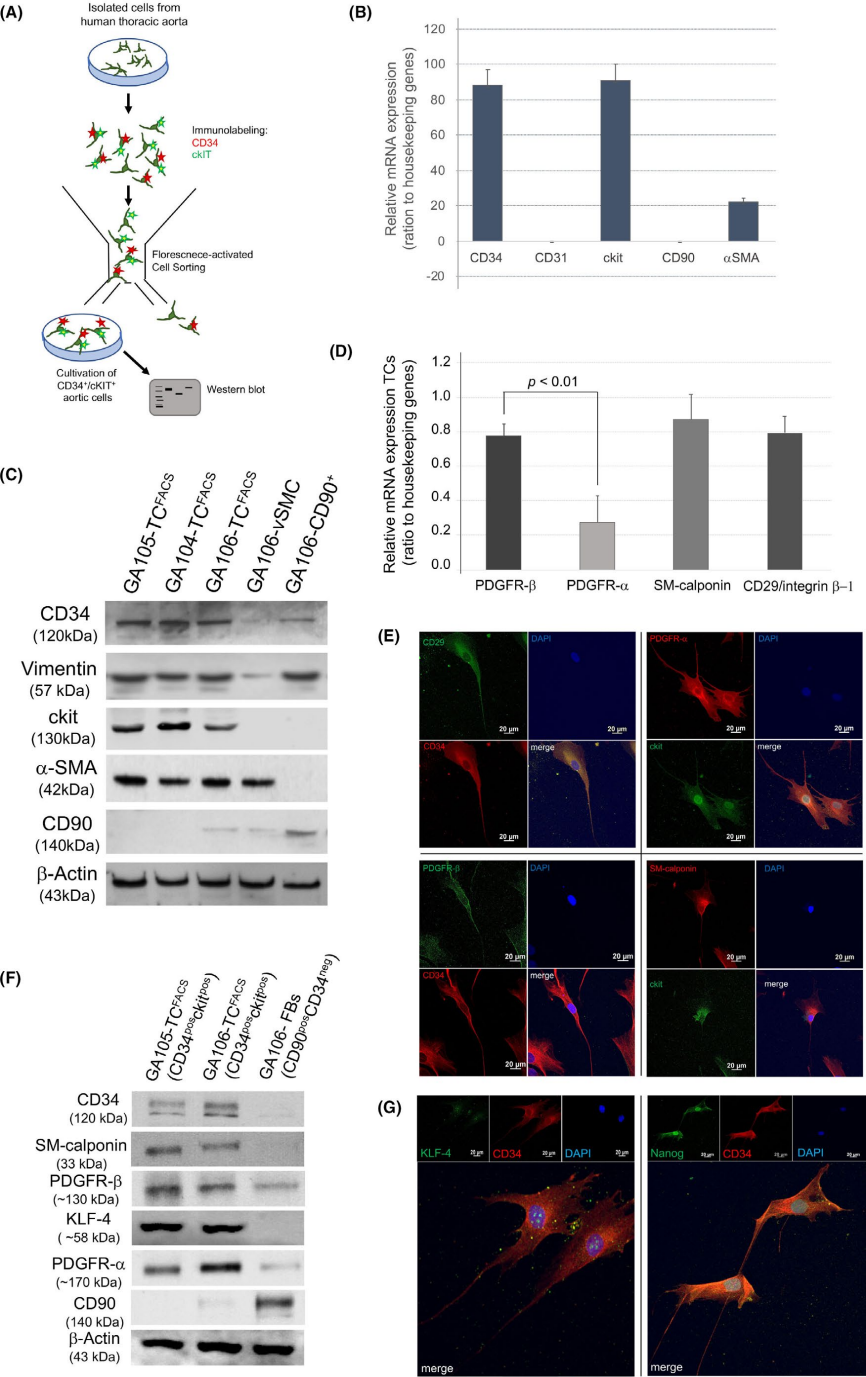
TC marker characterization. (A) Scheme of CD34^+^/c‐kit^+^ TC isolation. Primary aortic cells were labelled with CD34 and c‐kit, double‐positive cells were sorted by FACS and taken back into culture. For quality of FACS selection, CD34^+^/c‐kit^+^ TCs were controlled by mRNA expression (B) and verified by western blot (C). (B) Quantitative RT‐qPCR of FACS‐sorted TCs. Error bars represent the standard deviation of three independent experiments. (C) Western blot analysis of three individual aortic samples (GA104‐TC, GA105‐TC and GA106‐TC) after CD34^+^/c‐kit^+^‐cell sorting (FACS). αSMA^+^vSMCs (GA106‐vSMC) and CD90^+^ fibroblasts (GA106‐CD90^+^) isolated from HTA (sample GA106) were used as control. GAPDH was used as loading control; primary antibodies and the observed molecular weight (kDa) are given on the left. (D) mRNA expression profile of marker genes as indicated. Expression levels are presented. Error bars represent the SD based on three independent experiments. (E+F) Representative immunofluorescence and Western blot of TC marker proteins. GAPDH was used as loading control; primary antibodies and the observed molecular weight (kDa) are given on the left. (F) TCs also express stem cell marker proteins as shown by immunofluorescence. CD34 (red), Nanog (green) and KLF‐4 (green)

We next intended to characterize the molecular markers specific for aortic TCs by immunofluorescence and mRNA expression methods. First, we sorted primary HTA cells based on their expression of CD34 and c‐kit by FACS to isolate the TC population (Figure 4A). The CD34^+^/c‐kit^+^ cell population was confirmed by mRNA analysis and Western blot (Figure 4B and 4C) and cultivated for further analysis. Subsequently, we confirmed PDGFR‐α, PDGFR‐β, CD29/integrin β‐1 and SM‐calponin expression on sorted CD34^+^/c‐kit^+^‐TCs by mRNA (Figure 4D). Western blot analysis and immunofluorescence of cultured CD34^+^/c‐kit^+^‐TCs underlined the protein expression profile (Figure 4E and 4F). Human fibroblasts and vSMCs were used as control confirmed by Western blot (Figure 4C and 4F). With focus on stem cell markers involved in angiogenesis and vascular repair, we assessed the KLF‐4 and Nanog expression in CD34^+^/c‐kit^+^‐TCs. Both, KLF‐4 and Nanog expression, were detected in aortic TCs (Figure 4F and 4G).

### 3.5 CD34^+^/c‐kit^+^ TC’s microvesicles and exosomes contain angiogenic factors

**FIGURE 5:**
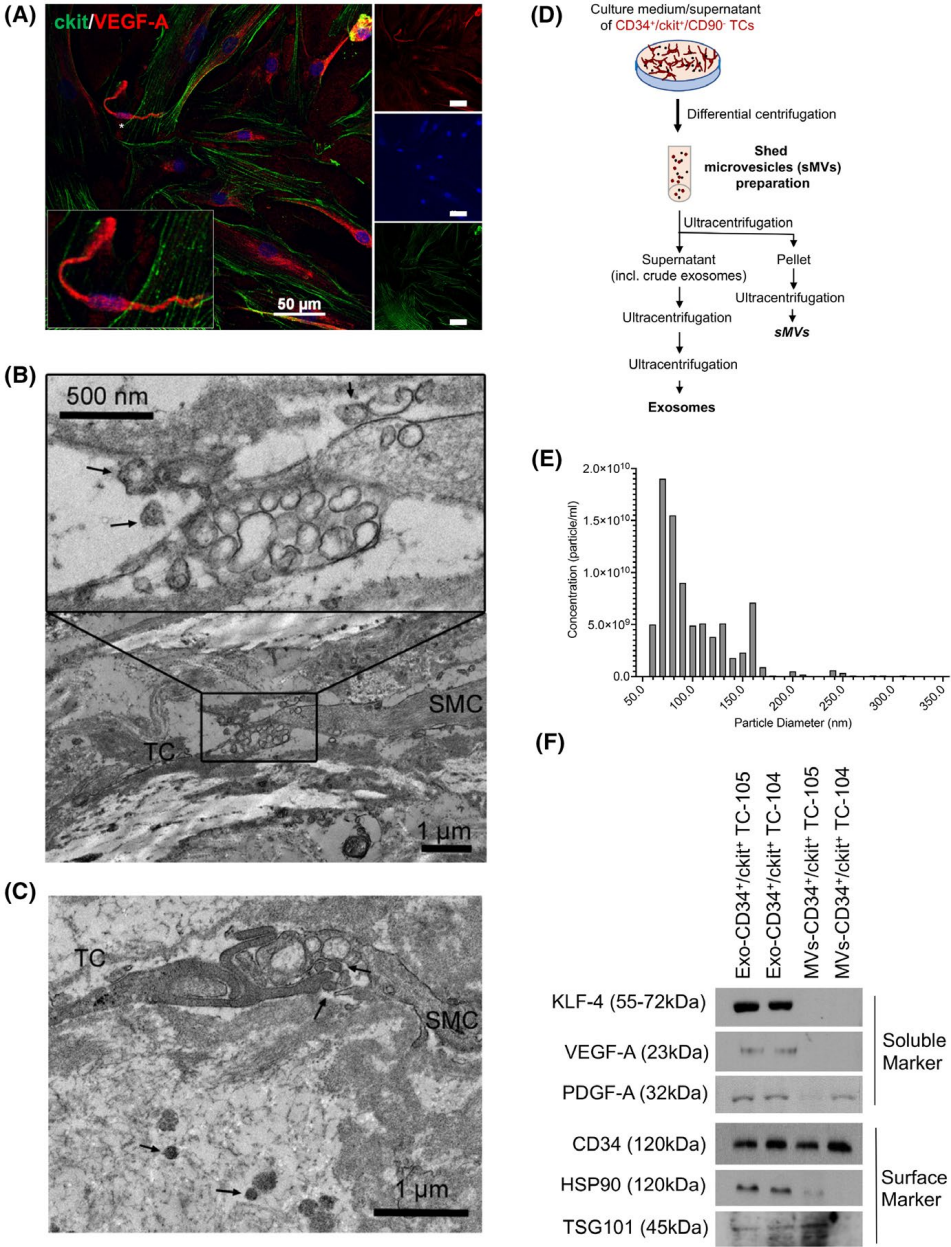
Characterization of exosomes/microvesicles. (A) Immunofluorescence labelling of c‐kit (red), VEGF‐A (green) and DNA (DAPI, blue). Primary TCs were identified by c‐kit positivity and showed intracellular anti‐VEGF‐A staining. The magnified cell (inset) is indicated by an asterisk. Images of single fluorescence channels are shown to the right. (B+D) Transmission electron microscopy of shed microvesicles (arrows) found next to the close connection of TC’s telopode and SMC. TC, telocyte; SMC, smooth muscle cell. (D) Scheme of exosome and sMV isolation from TC culture supernatant. (E) The quality, concentration (particle per ml) and particle diameter (nm) of exosomes were confirmed by qNano analysis (Izon instrument, UK). (F) Western blot analysis of exosome and MV preparations of two independently isolated aortic TCs (TC‐104, TC‐105). Exosome‐specific surface proteins (CD34, HSP90 and TSG101) as well as soluble factors (KLF‐4, VEGF‐A and PDGF‐A) were analysed. The respective molecular weight is given in brackets

TCs are known for their regenerative properties.^15^ Aortic cell homeostasis, including repair and regeneration, depends on angiogenic factors as VEGF.^28^, ^30^ Thus, we tested sorted CD34^+^/c‐kit^+^ TCs for VEGF‐A expression and confirmed very high levels (Figure 5A). Inter‐cellular communication by releasing extracellular vesicles has been demonstrated to be another function of TCs.^11^, ^31^ VEGF‐A is a soluble factor^32^; hence, we went on to test the hypothesis that TCs may be involved in aortic tissue homeostasis by shedding microvesicles (sMVs) and exosomes containing angiogenic factors such as VEGF‐A.

TEM analysis showed TCs, Tps and their podoms containing mitochondria, rough endoplasmic reticulum and specific vesicles (Figures 2, 5B, 5C). Putative extracellular vesicles were found throughout the aortic tissue but at much higher density in the immediate vicinity of Tps (Figure 5B and 5C). We isolated exosomes and MVs by ultra‐high centrifugation (Figure 5D) and analysed the quality and surface markers of isolated vesicles by qNano and Western blot (Figure 5E and 5F). The identity of exosomes and sMVs was confirmed by the presence of cell surface proteins such as HSP90 and TSG101.^22^ Moreover, we found the CD34 and KLF‐4 in isolated exosome and microparticles, which correlates with the expression of those proteins in TCs. With respect to angiogenic markers, we detected VEGF‐A and PDGF‐A in the exosomal fraction. In summary, our study reveals the presence of TCs in the HTA and provides further insights towards the goal of understanding this tissue. We suggest that TCs communicate with their environment via exosomes containing angiogenic factors such as VEGF‐A, PDGF‐A and KLF‐4.

## 4 DISCUSSION

Our results demonstrate the presence of TCs in the human aortic wall. TCs are mainly found in the tunica adventitia of HTA and form a network amongst themselves as well as contacts with neighbouring cell types. The morphological features of which strongly suggest a communicative function via direct cell contacts and paracrine MVs/exosome release. Aortic TC culture and cell sorting offered a unique method of investigating factors that may influence the normal function of TCs, interaction between TCs and aortic SMCs. Whilst previous studies of small and medium arterial vessels only described the existence of TCs by histopathological and TEM methods, our study focussed on marker proteins of TCs and the modes of inter‐cellular communication.

To date, CD34 and vimentin were identified as markers for vascular TCs in middle‐sized arteries.^12^ The mesenchymal cell marker vimentin is also expressed in vSMCs and fibroblasts. Similar to TCs, endoneural fibroblasts are also CD34 positive.^15^, ^33^ Our study shows that a combination of six marker proteins, CD34, c‐kit, PDGFR‐α/‐β, vimentin and CD29/integrin β‐1, can and should be used to identify and characterize aortic TCs. The additional identification of angiogenic and stem cell factors (VEGF‐A, Nanog, KLF‐4 and PDGF‐A) expressed by aortic TCs underpins the potential role of TCs in aortic tissue homeostasis of healthy individuals. The combination of CD34 positivity and CD31 negativity is widely accepted to be a typical marker human adventitial vascular wall‐resident stem/progenitor cells (VRS/Pcs).^34^, ^35^, ^36^ VRS/Pcs were reported to maintain homeostasis of the tunica media by differentiating into SMC‐like cells and migrating into the outer layer of the media.^37^ A CD34^+^CD31^−^‐ VRS/Pc population co‐expressing the haematopoietic marker c‐kit was found in the mouse.^38^ Concordantly, we found TC/SMC‐hybrid cells, that is CD34^+^TCs which express c‐kit, vimentin but also low levels the SMC marker proteins SM‐calponin and αSMC. This suggests that these TC/SMC‐hybrid cells might be TCs having migrated into the media to influence ECM and SMC differentiation in case of vascular damage by releasing functional vesicles.

Previous studies suggested a communication between TCs and SMCs by gap junctions or by paracrine mechanisms such as exosome release.^1^, ^9^, ^39^ Additionally, TCs were shown to not only release exosomes and ectosomes, but also multivesicular cargos.^40^ TCs were described to modulate stem cells by secretion of soluble factors.^41^ Thus, TCs seem to be able to induce and influence both, healthy homeostasis and pathological changes in aortic cell/ECM composition. For instance, in case of vascular injury or damage (eg continuous cell stress), SMCs are be modulated (or dedifferentiate) from a mature ‘contractile’ to a less differentiated ‘synthetic’ phenotype.^42^, ^43^ KLF‐4 has been shown to play a crucial role in regulating SMC (de‐)differentiation in aortic tissue.^44^, ^45^, ^46^ We found that KLF‐4 was highly expressed in aortic TCs. This finding provides the first indication of a possible pericyte role in angiogenic processes. The release of exosomes with the stem cell factor KLF‐4, as well as the CD34^+^/c‐kit^+^/vimentin^+^/αSMA^+−^ TC subtypes, seems to be involved in keeping the balance of a physiological SMCs contractile phenotype.^46^ c‐kit was recently described to play a crucial role in TCs and their function in tissue regeneration.^2^, ^31^, ^47^ It was suggested that the c‐kit‐positive TC subtype contributes to cell maintenance and homeostasis and may participate in repair processes.^48^ We therefore propose a corresponding function of c‐kit‐positive TCs in the human aorta. Excessive stem cell factor expression/secretion of TCs could lead to the dedifferentiated synthetic phenotype of vSMCs and pathological ECM remodelling. Moreover, VEGF‐A appears to be strongly associated with aortic tissue remodelling, and later, aneurysm rupture.^49^ Thus, VEGF‐A and KLF‐4 expressed in aortic TCs should have essential functions for tissue regeneration of the aorta. An imbalance between both factors might have fatal consequences leading to aortic aneurysms.

In conclusion, our data provide novel evidence for the existence of TCs in human aorta, which may be relevant in cell homeostasis, regeneration and tissue remodelling. Further functional studies are needed to investigate the response of aortic TCs to oxidative stress and blood pressure‐related increased wall tension in pathological dilatation of human aorta. Stress‐ and immunogenic‐induced TC cell proliferation and therefore increased exosome release of soluble stem cell factors such as PDGF‐A and KLF‐4 may initiate an incessant cascade of remodelling steps in aneurysm pathology. Thus, PDGF‐A and KLF‐4 seem to play a critical role in aortic aneurysm formation via induction of pathological phenotype switching of SMCs and also lead to altered extracellular matrix composition.

## CONFLICTS OF INTEREST

The authors confirm that there are no conflicts of interest.

## AUTHOR CONTRIBUTIONS

**Thomas Aschacher:** Conceptualization (lead); Data curation (lead); Formal analysis (equal); Funding acquisition (lead); Investigation (equal); Methodology (equal); Project administration (lead); Resources (equal); Supervision (lead); Validation (equal); Visualization (equal); Writing‐original draft (lead); Writing‐review & editing (lead). **Katy Schmidt:** Conceptualization (supporting); Data curation (supporting); Formal analysis (supporting); Investigation (equal); Methodology (equal); Resources (supporting); Validation (supporting); Visualization (supporting); Writing‐review & editing (supporting). **Olivia Aschacher:** Conceptualization (supporting); Data curation (supporting); Project administration (supporting); Visualization (supporting); Writing‐original draft (supporting); Writing‐review & editing (supporting). **Eva Eichmair:** Data curation (supporting); Formal analysis (supporting); Investigation (supporting); Methodology (supporting); Validation (equal); Visualization (supporting). **Ulrike Baranyi:** Data curation (supporting); Investigation (supporting); Methodology (supporting); Resources (supporting); Validation (supporting). **Andreas Spittler:** Investigation (supporting); Resources (supporting); Validation (supporting). **Bernhard Winkler:** Conceptualization (supporting); Data curation (supporting); Resources (supporting); Writing‐review & editing (supporting). **Martin Grabenwoeger:** Conceptualization (supporting); Resources (supporting); Writing‐review & editing (supporting). **Florian Karl Enzmann:** Conceptualization (supporting); Data curation (supporting); Formal analysis (supporting); Validation (supporting); Writing‐original draft (supporting); Writing‐review & editing (supporting). **Barbara Messner:** Data curation (supporting); Formal analysis (supporting); Investigation (supporting); Methodology (supporting); Resources (equal); Validation (supporting); Writing‐review & editing (supporting). **Julia Riebandt:** Investigation (supporting); Resources (supporting); Writing‐review & editing (supporting). **Guenther Laufer:** Funding acquisition (supporting); Project administration (supporting); Resources (supporting); Writing‐review & editing (supporting). **Michael Bergmann:** Conceptualization (supporting); Resources (supporting); Validation (supporting); Writing‐review & editing (supporting). **Marek Ehrlich:** Conceptualization (supporting); Funding acquisition (supporting); Project administration (supporting); Resources (supporting); Supervision (supporting); Writing‐review & editing (supporting).
